# A nationwide analysis of the excess death attributable to diabetes in Brazil

**DOI:** 10.7189/jogh.10.010401

**Published:** 2020-06

**Authors:** Paula A Bracco, Edward W Gregg, Deborah B Rolka, Maria Inês Schmidt, Sandhi M. Barreto, Paulo A Lotufo, Isabela Bensenor, Dora Chor, Bruce B Duncan

**Affiliations:** 1 Postgraduate Program in Epidemiology, School of Medicine and Hospital de Clínicas de Porto Alegre, Universidade Federal do Rio Grande do Sul, Porto Alegre, Rio Grande do Sul, Brazil; 2 Department of Diabetes and Cardiovascular Disease Epidemiology, School of Public Health, Imperial College London, UK; 3 National Center for Chronic Disease Prevention and Health Promotion, Division of Diabetes Translation, Center of Disease Control, Atlanta, Georgia, USA; 4 Department of Preventive and Social Medicine, School of Medicine, Universidade Federal de Minas Gerais, Belo Horizonte, MG, Brazil; 5 Department of Internal Medicine, School of Medicine, University of Sao Paulo, São Paulo, SP, Brazil; 6 Department of Epidemiology and Health Quantitative Methods, National School of Public Health, Oswaldo Cruz Foundation, Rio de Janeiro, RJ, Brazil

## Abstract

**Background:**

Data on mortality burden and excess deaths attributable to diabetes are sparse and frequently unreliable, particularly in low and middle-income countries. Estimates in Brazil to date have relied on death certificate data, which do not consider the multicausal nature of deaths. Our aim was to combine cohort data with national prevalence and mortality statistics to estimate the absolute number of deaths that could have been prevented if the mortality rates of people with diabetes were the same as for those without. In addition, we aimed to estimate the increase in burden when considering undiagnosed diabetes.

**Methods:**

We estimated self-reported diabetes prevalence from the National Health Survey (PNS) and overall mortality from the national mortality information system (SIM). We estimated the diabetes mortality rate ratio (rates of those with vs without diabetes) from the Brazilian Longitudinal Study of Adult Health (ELSA-Brasil), an ongoing cohort study. Joining estimates from these three sources, we calculated for the population the absolute number and the fraction of deaths attributable to diabetes. We repeated our analyses considering both self-reported and unknown diabetes, the latter estimated based on single point-in-time glycemic determinations in ELSA-Brasil. Finally, we compared results with diabetes-related mortality information from death certificates.

**Results:**

In 2013, 65 581 deaths, 9.1% of all deaths between the ages of 35-80, were attributable to known diabetes. If cases of unknown diabetes were considered, this figure would rise to 14.3%. In contrast, based on death certificates only, 5.3% of all death had diabetes as the underlying cause and 10.4% as any mentioned cause.

**Conclusions:**

In this first report of diabetes mortality burden in Brazil using cohort data to estimate diabetes mortality rate ratios and the prevalence of unknown diabetes, we showed marked underestimation of the current burden, especially when unknown cases of diabetes are also considered.

Diabetes mellitus, given its growing prevalence and its diverse ensuing morbidity, is one of the world’s major chronic threats to public health. With a global prevalence in 2017 estimated as 8.8%, diabetes currently affects approximately 425 million individuals between 20 and 79 years worldwide [1]. The prevalence of diabetes in Brazil, as in most countries of the world, has increased dramatically. Self-reported medical diagnosis of diabetes in Brazilian state capital cities increased from 5.5% in 2006 to 8.9% in 2016 [2]. Additionally, between 2010 and 2015, estimated diabetes prevalence increased approximately 450 000 cases per year [3].

Although prevalence data are readily available or can be estimated for most countries in the world, data on the mortality burden and excess deaths attributable to diabetes are sparse and frequently unreliable, particularly in low and middle-income countries where an estimated 75% of diabetes cases live [4]. Mortality information systems in these countries are frequently incomplete and inaccurate. Even when complete, the subjective assignment of diabetes as a cause of death on death certificates likely leads to underestimates of the diabetes mortality burden [5]. For these countries, the mortality burden could be worse than in high-income countries, as fewer resources are available to treat the disease and its complications. A recent prospective study in Mexico highlighted this potential, concluding that the rate of death from any cause among those with diabetes was close to four times that of those without, and that the excess risk associated with self-reported diabetes accounted for 30% of all deaths [6].

Given the limitations of death certificate data and that estimates of the diabetes mortality burden in Brazil to date have relied on death certificates, they are of questionable validity. Epidemiologic mortality data from prospective cohorts following individuals with and without diabetes can be used as an alternative approach to better estimate a disease mortality burden, allowing the calculation of the number of excess deaths attributable to the disease, ie, the deaths due to the increased mortality rate among those with the disease and that could be potentially preventable if the mortality rate among those with the disease were the same as those without [7].

Thus, to evaluate the impact of diabetes on excess mortality in Brazil, we combined cohort data with national prevalence and death statistics to calculate the absolute number of deaths attributable to diabetes, potentially preventable if the mortality rate between those with and without diabetes were the same. Specifically, our aim was to calculate the proportion of total deaths that were due to diabetes for the year 2013 by employing a nationwide analysis combining longitudinal data from a large Brazilian multicenter study with national mortality statistics and prevalence estimates of known and unknown diabetes.

## METHODS

This study models data from three sources – a prospective Brazilian cohort, a nationally representative cross-sectional survey and official compiled data on mortality statistics derived from analyses of nationwide censuses.

### Data sources

Our estimates of diabetes prevalence for the Brazilian population are based on the 2013 National Health Survey (Pesquisa Nacional de Saúde, or PNS). Briefly, the PNS, as described in greater detail elsewhere [8,9], is a nationwide, household-based survey with multistage, probability sampling designed to produce reliable national estimates. In each household, one individual (aged 18 years or more) was selected, with equal probability among the adult residents, to answer personal questions about morbidity and lifestyle. For this analysis, we selected only individuals between 35 and 80 years with information on diabetes status, resulting in a sample of 64 308 participants. Survey weights were applied to account for the complex sampling design and to make estimates representative of the national non-institutionalized population of Brazil. Self-reported diabetes prevalence was assessed by the question “*Has a doctor ever told you that you have diabetes?*”. Then, to estimate the absolute number of people with diabetes for each gender and in each age group between 35 and 80 years, we multiplied the respective prevalence by the population projection for the year 2013 of the Brazilian Institute of Geography and Statistics [10].

Mortality rates for people with and without diabetes are not available from representative national sources. Thus, to estimate these rates, we first obtained the mortality rate ratio between individuals with and without diabetes from the Brazilian Longitudinal Study of Adult Health (ELSA-Brasil). ELSA-Brasil is a multicenter cohort study funded by the Brazilian government and designed primarily to investigate the causes and consequences of diabetes and cardiovascular diseases. The cohort comprised of 15 105 civil servants, aged 35 to 74 years at baseline (2008-2010), enrolled at universities or research institutions in six cities of three different regions of Brazil [11,12]. ELSA-Brasil was approved by institutional review boards at each of its centers; all study subjects gave written informed consent prior to their participation.

Known (self-reported) diabetes was accessed similarly at baseline in ELSA with the question “*Has a doctor ever told you that you have diabetes?*” ELSA-Brasil participants were followed with an annual telephone interview until July 2018 to assess mortality. In addition, blood samples were collected at baseline to perform laboratory tests. We excluded 16 participants with missing values for diabetes and 239 with missing values for any covariate used in modeling, producing a final data set of 14 850 participants and a total of 480 deaths. We used 2013 age and sex-specific Brazilian overall population mortality rates from the National Institute of Geography and Statistics for ages 35 to 80.

### Statistical analysis

We estimated diabetes prevalence from PNS by sex and age between 35-80 years by logistic regression, including a quadratic term for age. We used SAS SUDAAN to account for the survey sample design and to produce the weighted average marginal estimates. We estimated the mortality rate ratio for diabetes for each sex and age (35-74 years) from the ELSA-Brazil cohort with Cox regression, adjusting for body mass index (BMI), waist circumference, race/color, schooling, income and smoking status, and including interactions terms for age, diabetes and sex. The mortality rate ratio was then extrapolated for ages 75 to 80. We tested the goodness-of-fit of our models in comparison with the null hypothesis through the Likelihood test. In addition, we analyzed the proportional hazard assumption for each variable on our Cox regression model based on weighted Schoenfeld residuals.

We applied the mortality rate ratio to the overall male and female Brazilian mortality rates to obtain the age and sex-specific mortality rates among individuals with and without diabetes. We then used these mortality rates together with the number of people with diabetes in each age and gender strata to estimate the excess deaths due to diabetes in that strata. In addition, we calculated the population-attributable fraction (PAF) as the proportion of total deaths attributable to diabetes for the year 2013, following the method and detailed formulas of Jacobs et al., which were designed for use in situations in which nationwide mortality rates for individuals with and without diabetes are not available [13,14].

To investigate the influence of unknown (undiagnosed) diabetes on our results, we recalculated the diabetes mortality rate ratio and the mortality rates considering as diabetes cases in the ELSA-Brasil sample not only those who self-reported diabetes, but who also met, at a single point in time, at least one of the laboratory criteria for diagnosis (fasting glucose ≥126 mg/dL (7.0 mmol/L) or 2-hour glucose ≥200 mg/dL (11.1 mmol/L) or glycated hemoglobin (HbA1c)> 6.5% (48 mmol/mol). In addition, we also increased our estimates of diabetes prevalence for the Brazilian population based on the proportion of undiagnosed diabetes by age group observed in ELSA-Brasil. We compared the proportion of undiagnosed diabetes among men and women in each age group using Fisher Exact Test. We then recalculated excess deaths and the PAF.

We also compared our results with estimates generated by calculating the proportion of deaths due to diabetes from death certificate information available on the Brazilian Mortality Information System: first, when diabetes was reported as the main cause and second, permitting any mention of diabetes on the death certificate, as opposed to standard underlying cause coding approaches.

All analysis and graphs were performed in SAS 9.4 or SAS-SUDAAN 9.3 (SAS Institute, Cary NC, USA). We considered significant *P-*values <0.05.

## RESULTS

**Figure 1:**
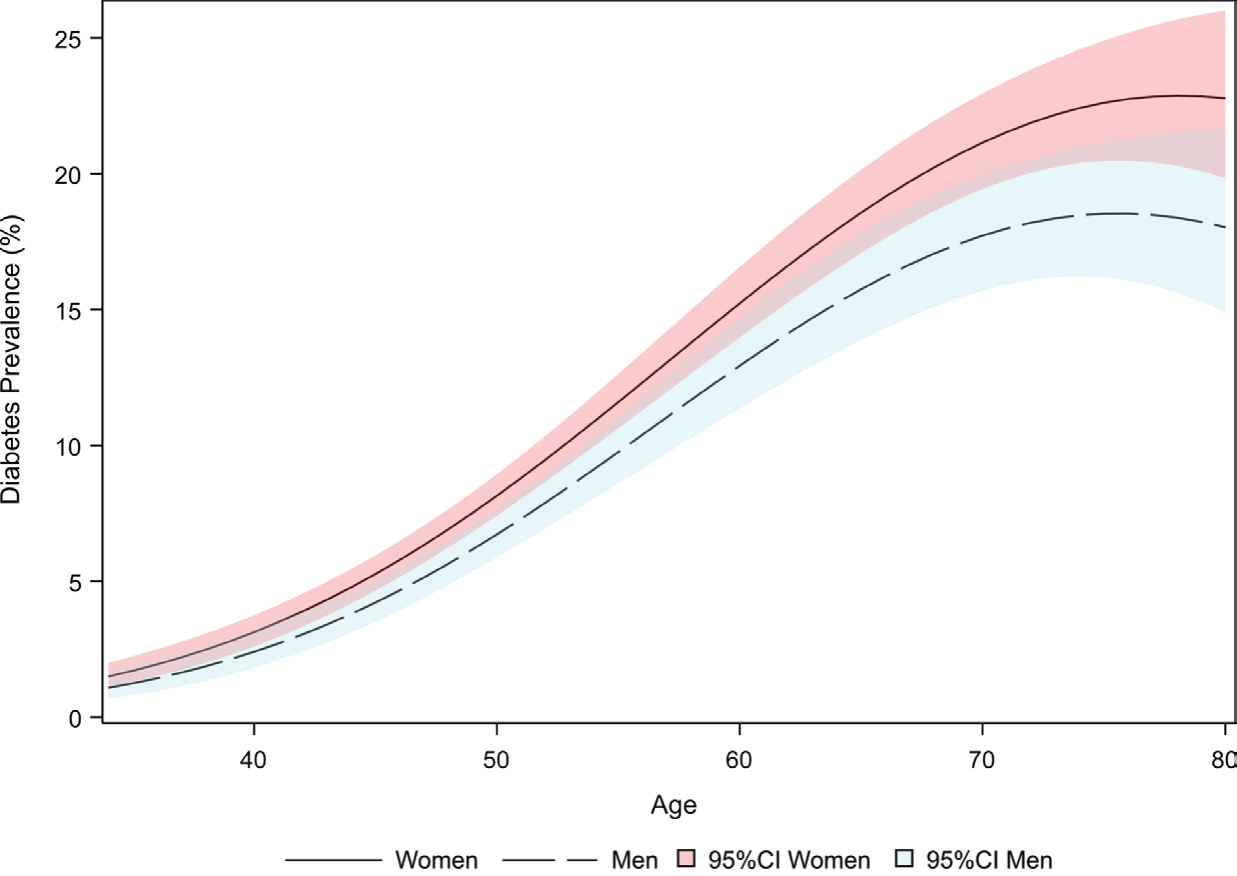
Prevalence of diagnosed diabetes (%) for Brazilian men (dashed line; blue) and women (solid line; red). National Health Survey (Pesquisa Nacional de Saúde, PNS), Brazil, 2013.

The prevalence of known diabetes in the National Health Survey was higher in women than in men (**Figure 1**), with the highest prevalence occurring around age 75 (approximately 23% for women and 18% for men). The proportion of undiagnosed diabetes estimated from ELSA-Brasil is similar for both sexes (50.2% in women and 51.5% in men; *P* = 0.53). The highest proportion of undiagnosed cases occurs in women aged 70-79 (61.9%) and men aged 60-69 (61.5%) (**Table 1**).

**Table 1:** Proportion (%) of undiagnosed diabetes cases by sex and age group in ELSA-Brasil, 2008-2010

Age group	Women (%)	Men (%)	*P-*value
35-49	40.9	43.0	0.63
50-59	49.5	50.6	0.76
60-69	56.9	61.4	0.25
70-79	61.9	53.2	0.33
**Total**	50.2	51.5	0.53

*P-values are calculated from Fisher Exact Test for the difference between men and women.

**Figure 2:**
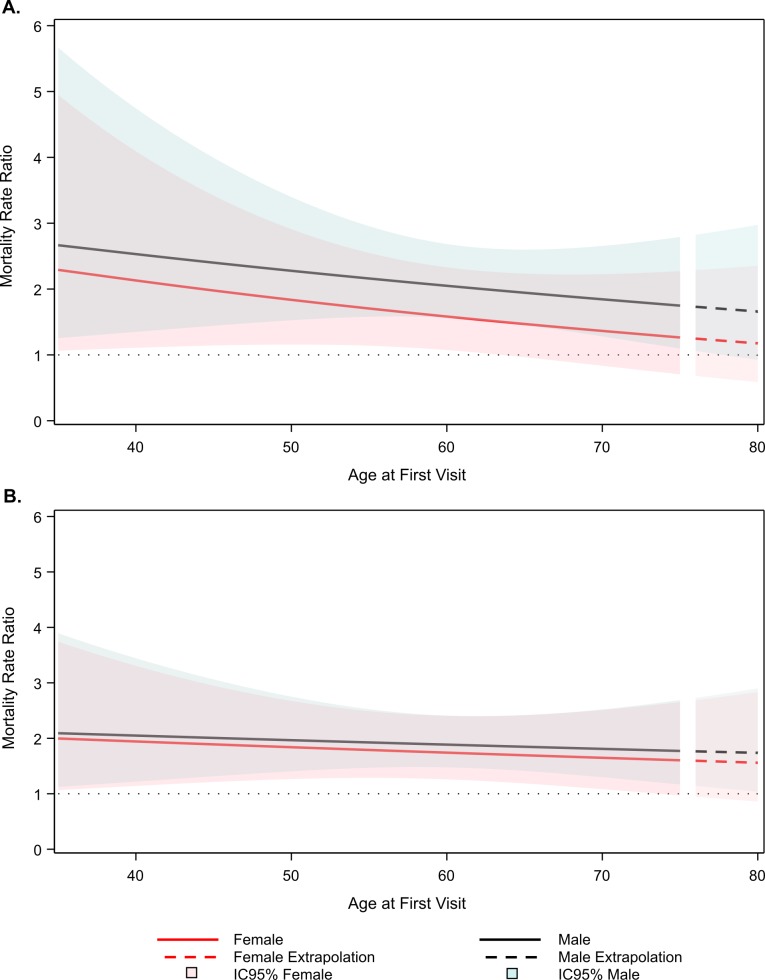
Adjusted mortality rate ratio (diabetes vs non-diabetes) (adjusted for ethnicity, educational level, income, smoking status, BMI and waist circumference). **Panel A**. Self-reported. **Panel B**. Self-reported plus undiagnosed diabetes. Men – black line, for men, blue confidence zone, women – red line, pink confidence zone. Estimates between ages 75 and 80 years old (small dashed lines) are extrapolations. ELSA-Brasil, 2008-2018.

**Figure 3:**
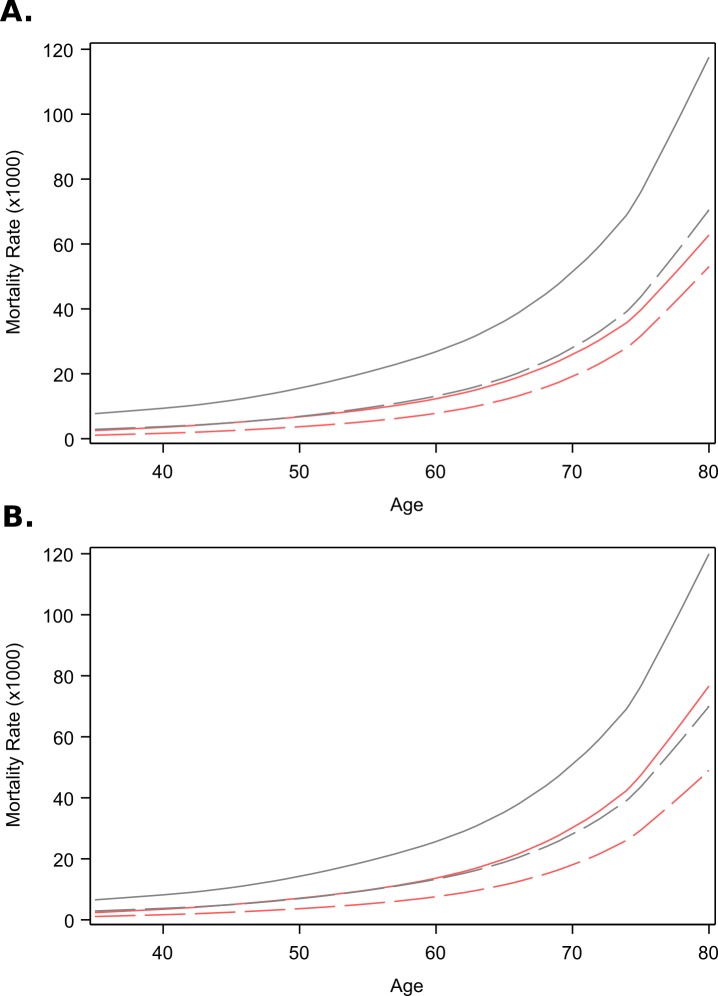
Mortality rates. **Panel A**. Self-reported. **Panel B**. Self-reported plus undiagnosed diabetes. Men – black lines, women – red lines. Dashed lines represent mortality of those without diabetes. Brazil, 2013.

The adjusted mortality rate ratio for self-reported diabetes among ELSA-Brasil participants decreased with age, from 2.38 (95% confidence interval (CI) = 1.14-5.01) at age 35 to 1.42 (95% CI = 0.877-2.29) at age 70 in women, and from 2.76 (95% CI = 1.33-5.70) at age 35 to 1.88 (95% CI = 1.32-2.68) at age 70 in men (**Figure 2**, panel A). At age 80, the estimated adjusted diabetes mortality rate ratios were 1.22 (95% CI = 0.618-2.41) and 1.69 (95% CI = 0.96-2.98) respectively. Values are extrapolated after age 75 and presented with a dashed line. The mortality rates for people with and without diabetes, obtained by combining overall mortality rates with the diabetes mortality rate ratio, were consistently higher in men than in women in all ages. Interestingly, in men without diabetes the mortality rate reached that of women with diabetes (**Figure 3**, panel A).

When undiagnosed diabetes cases were considered, the mortality rate ratios decreased slightly, changed less across the age range, and were similar for men and women (**Figure 2**, panel B).

Combining the above rates of self-reported diabetes with mortality rate ratios and national population estimates, we calculated 65 581 deaths in 2013 as being attributable to diagnosed diabetes (**Table 2**) – 9.1% of all deaths for that year. Excess deaths, in absolute numbers and as a fraction of the total deaths, were higher in men (44 591, PAF 10.5%) than in women (20 990, PAF 7.2%). In age-stratified analyses, the PAF was 4.9% among those aged 35-49, and peaked at 11.3% in those aged 60-69. When accounting for undiagnosed diabetes, the large increase in prevalence and the smaller decrease with age in the mortality rate ratio resulted in an increase in the excess deaths due to diabetes, with an estimate of 102 350 deaths attributable to diagnosed diabetes and a corresponding PAF of 14.3%. In this scenario, although the absolute number of excess deaths is still higher in men (58 228) than in women (44 122), the proportion of total deaths attributable to diabetes is slightly higher in women (15.1%) than in men (13.7%).

**Table 2:** Comparison of the number of excess deaths and percent of deaths attributable to diabetes when considering A) only diagnosed diabetes, B) additionally undiagnosed diabetes ascertained by laboratory testing and C) any mention of diabetes on the death certificate, Brazil 2013

	Women			Men			Total		
**Diabetes Prevalence, % (CI 95%)**	Excess deaths among people with diabetes,* N	**PAF†, %**	**Diabetes Prevalence, % (CI 95%)**	Excess deaths among people with diabetes,*N	**PAF†, %**	**Diabetes Prevalence, % (CI 95%)**	Excess deaths among people with diabetes,*N	**PAF†, %**
**A. Diagnosed diabetes – Age group:**									
35-49	4.17 (3.61-4.82)	2062	4.73	3.30 (2.67-4.11)	4434	4.97	3.74 (3.14-4.47)	6496	4.89
50-59	11.28 (10.33-12.30)	4643	8.05	9.48 (8.34-10.75)	10 126	10.32	10.38 (9.33-11.52)	14 769	9.48
60-69	18.14 (16.69-19.70)	7072	8.73	15.37 (13.55-17.38)	14 993	13.05	16.76 (15.12-18.54)	22 065	11.26
70-79	22.32 (20.17-24.61)	7213	6.55	18.29 (15.92-20.93)	15 039	12.27	20.30 (18.05-22.77)	22 252	9.56
**Total**	12.89 (11.69-14.19)	20990	7.18	10.69 (9.30-12.27)	44 591	10.50	11.79 (10.49-13.23)	65 581	9.14
**B. Diagnosed and undiagnosed diabetes**	20.02 (18.17-22.03)	44122	15.09	16.43 (14.32-18.89)	58 228	13.71	18.21 (16.21-20.42)	102 350	14.27
**C. Death certificate:**									
Main cause	-	-	6.03	-	-	4.27	-	-	5.27
Contributing cause	-	-	11.84	-	-	8.51	-	-	10.39

PAF – population attributable fraction, CI – confidence interval

*Absolute number of deaths within people with diabetes that could have been avoided if the mortality rate in people with diabetes were equal to the mortality rate of people without diabetes.

†The percent of the overall total number of deaths (people with and without diabetes) attributable to diabetes.

The diabetes mortality burden estimated from death certificate data in Brazil´s national mortality system was 5.3% when diabetes was the underlying cause and 10.4% when any mention of diabetes appeared on any line of the death certificate. Both estimates were higher in women than in men (**Table 2**).

## DISCUSSION

This is the first nationwide estimate of excess deaths attributable to diabetes in Brazil, and one of the few estimates of this burden in low- and middle-income countries. Compared to those without diabetes, mortality of those with known diabetes was 2.76 and 1.88 times higher among middle-aged and elderly men; and 2.38 and 1.42 times higher among middle-aged and elderly women, respectively. In 2013, 65 581 deaths, approximately 9% of all deaths between the ages of 35-80, could have been prevented if the mortality rate were the same between those with and without known diabetes. Given the large fraction of total diabetes cases ascertained only by laboratory measurements in ELSA, if excess deaths among these cases of unknown diabetes were also considered, this figure would rise to 14%.

In contrast, the Global Burden of Disease Study estimated that, in 2013, only 5.1% of all deaths in Brazil were due to diabetes among individuals between 50 and 69 years old, and only 4.2% when considering all ages [15]. These estimates are largely based on data of the Brazilian Mortality Registry (Sistema de Informações sobre Mortalidade, or SIM), in which 5.3% of death certificates indicated diabetes as the underlying cause. Using death certificates as the principal source of mortality data to guide Brazilian public health decisions thus estimates a diabetes mortality burden approximately 50% less than that which we report. The Brazilian vital registration system has improved considerably in recent decades [16]. However, estimating diabetes mortality burden from death certificate data are challenging and considerably less sensitive in detecting deaths attributable to diabetes than data sources such as administrative records or surveys [5]. This difference largely results from the fact that most individuals with diabetes have multiple comorbidities and complications that contribute to their cause of death [17]. As only one main cause is permitted when following the International Classification of Diseases, other conditions, especially cardiovascular and renal diseases, are often chosen, leaving an unrecognized contribution of diabetes. Given the complications in estimation due to the multicausality of diseases and deaths, our cohort-based analysis, which permits a direct contrast in risk of death between those with and without diabetes, provides a more appropriate estimate.

Of note, if we consider as diabetes-related deaths those with mention of diabetes on any line of the death certificate [18], the percentage of 2013 diabetes-related deaths between 35 and 80 years rises to 10.4%, close to the attributable fractions estimated based on our cohort data. This similarity demonstrates that, in the absence of cohort data, the fraction of deaths having diabetes listed anywhere on the death certificate appears to be a more reasonable estimate of the true diabetes mortality burden.

For 2013, the International Diabetes Federation, applying a similar analysis, estimated that 226 371 deaths (11.6% of total deaths) were attributable to diabetes in adults of Central and South America aged between 20-79 years [19]. These estimates, although close to our results, are an approximation for the whole region and are based on sex- and age-specific relative risks of death between individuals with and without diabetes derived from United States (US) National Health and Nutrition Examination Surveys (NHANES) performed between 1971 and 1993 [20]. As NHANES is representative of the United States population, our use of relative diabetes mortality obtained from a Brazilian cohort allows a more valid estimate for the Brazilian diabetes mortality burden.

The majority of studies reporting excess deaths based on relative risk or the rate ratio of mortality for diabetes comes from high-income countries. A meta-analysis of 97 prospective cohorts in 25 countries (all but two of them high-income) showed that persons with diabetes had almost twice the rate of death from any cause as those without the disease [21]. In the United States, for the period of 1999-2010, the estimated hazard ratio for those with vs without diabetes, aged 34 to 80 years old, was 2.0 (95% CI = 1.75-2.28) for self-reported diabetes and 1.88 (95% CI = 1.63-2.16) for diabetes diagnosed by HbA1c criteria or self-reported use of an oral hypoglycemic agent or insulin [22]. As in our findings, the diabetes mortality hazard ratio (HR) observed for the United States also decreased with age. A similar pattern was described for the Swedish population included in their National Diabetes Register on or after 1998 and followed until 2011, with a diabetes mortality HR of 2.59 (95% CI = 2.27-2.96) for individuals younger than 55 years old and 1.03 (95% CI = 1.01-1.06) for those older than 75 years of age [23].

Attributable fractions similar to ours have been reported previously. A recent study from Germany, using insurance claims data to estimate diagnosed type 2 diabetes of individuals between 40 and 99 years, concluded that approximately 16.4% of deaths occurring in 2010 could have been avoided if the mortality rate of those with diabetes equaled that of those without the disease [14]. From the NHANES data, the diabetes mortality PAF was 11.8% for self-reported diabetes in the US population aged 30 to 84 years and 11.7% when using HbA1c criteria [22].

Application of data from longitudinal studies in the calculation of national diabetes-related outcomes data are rare for low and middle-income countries. The only study we located, which shows recent results from Mexico, found considerably greater risks of death associated with diabetes than observed in high income countries, with the diabetes death rate ratio ranging from 5.4 (95% CI = 5.0-6.0) for individuals aged 35 to 59 years to 1.9 (95% CI = 1.8-2.1) for those 75 to 84 years old. Considering excess deaths, 30% of all deaths of Mexicans between ages 35 to 74 years were due to diabetes; this estimate increased to 35% when including undiagnosed diabetes [24]. A more favorable ratio in Brazil than in Mexico could be the result of decreases in diabetes mortality rates, as has been observed in death certificate data in Brazil over the last decade [25]. Additionally, we observed trends to lower mortality due to acute complications of diabetes from 1991 to 2010 [26]. These declines occurred in parallel to improvements of the Brazilian National Health System (SUS) coverage, especially relating to primary care, which provided greater access to medications such as antibiotics and insulin and to qualified care. Similar findings have been reported for many high-income countries [27-29].

The Mexican and Swedish studies reported virtually identical relative diabetes mortality rates for men and women and no significant interaction between sex and diabetes for all-cause mortality, respectively [23,24]. Although we also found no significant difference, we consistently found higher diabetes mortality rate ratios in men than in women for all ages, the difference being considerably wider when not including undiagnosed diabetes. A possible explanation for these findings is that milder cases are more likely to go undiagnosed in Brazilian men than in Brazilian women with diabetes.

Even though diabetes prevalence was higher in women, the percentage of total deaths due to diabetes was higher in men for self-reported diabetes, in contrast with the findings for the US population [22]. However, the decrease in the mortality rate ratio difference between sexes and across ages after accounting for undiagnosed diabetes resulted in a slightly higher PAF for women. This second finding is in accordance to what has been reported when analyzing death certificates, ie, that the diabetes mortality rate in Brazil in recent years has been higher in women than in men [25]. The higher rates for women can in part be due to the greater diagnosis of the disease in women, as they tend to seek more health services, thereby obtaining the diagnosis of diabetes more often than men [30]. The higher prevalence of diabetes in women results in their deaths representing a higher fraction of the total deaths, however it may suggest that our results could be underestimating the diabetes mortality burden in men.

Limitations to our study merit consideration. The ELSA cohort is not a representative sample of the entire Brazilian population, since it consists of university and research institute employees with stable jobs who, in comparison with the general population, have greater educational attainment, a greater proportion of white ethnicity, and higher income. However, in terms of self-reported diabetes, its results are similar to those found in representative surveys of the Brazilian state capitals [12]. Moreover, our estimates of diabetes mortality rate ratio are adjusted not only for age, sex, BMI, and central obesity, but also for ethnicity, smoking status, educational level, and income. Another possible limitation is that our estimation of unknown diabetes is based on one of several glycemic tests, some of which present large within-individual variability. Studies suggest that approximately 25% of those ascertained as having diabetes based on one laboratory determination will not be positive on retesting [31]. Thus, while these estimates may be accurate for “known diabetes plus those who would be ascertained on single testing as having diabetes”, they overestimate the PAF for known and unknown diabetes. However, even if only half of those ascertained with undiagnosed diabetes on single testing in ELSA in fact had unknown diabetes, the increase in estimated excess deaths and PAF would remain notable.

Within these limitations, ELSA provides accurate and necessary data to estimate the diabetes mortality burden. When coupled with nationally representative estimates of prevalence of diabetes and overall estimates of mortality rates, these data obviate the need to use death certificate cause of death information. In addition, it also allows us to differentiate the mortality rate ratio for self-reported diabetes alone and when accounting for undiagnosed diabetes. Therefore, even with our limitations, these results contribute greatly to the scarce information published about diabetes burden in low- and middle-income countries.

## CONCLUSION

In conclusion, combining longitudinal cohort data of ELSA-Brasil with national diabetes prevalence data from the PNS and national mortality data, we calculated the absolute number of excess deaths for individuals with diabetes in Brazil in 2013. We believe that this is the first report of the diabetes mortality burden in Brazil based on means other than death certificates, which underestimate the importance of diabetes as a cause of death. We found that, despite a recent lowering in diabetes mortality rates, the diabetes mortality burden is quite high in Brazil – 9.1% when considering only self-reported diabetes, and 14.3% when adding undiagnosed diabetes ascertained with a single determination. These data emphasize the importance of greater actions by Brazilian society and governments to confront the ongoing obesity and diabetes epidemics.
